# An empirical study on the impact of executive compensation incentives on the performance of Chinese pharmaceutical firms

**DOI:** 10.3389/fpubh.2026.1962847

**Published:** 2026-10-23

**Authors:** Huihui Zhang, Boyi Xu, Su Wang, Yuwen Chen

**Affiliations:** 1 School of Business Administration, Shenyang Pharmaceutical University, Shenyang, China; 2 Drug Regulatory Research Base of NMPA, Research Institute of Drug Regulatory Science, Shenyang Pharmaceutical University, Shenyang, China

**Keywords:** executive compensation incentives, financing constraints, firm growth, firm performance, pharmaceutical manufacturing firms

## Abstract

As the “Healthy China” initiative continues to gain momentum, the pharmaceutical manufacturing industry is entering a pivotal phase of shifting from quantitative expansion toward innovation-driven growth. Senior executives, as the primary architects of firm strategic decisions, wield compensation incentives that profoundly affect resource allocation efficiency and long-term firm performance. Drawing on a panel dataset of A-share listed pharmaceutical firms on the Shanghai and Shenzhen stock exchanges over the period 2013–2024, this study empirically examines the impact of executive remuneration incentives on firm performance. Moreover, it investigates the mediating role of financing constraints and the moderating effect of firm growth, while also accounting for regional heterogeneity in the observed relationships. Our empirical findings yield several key insights. First, executive compensation incentives exert a significantly positive influence on firm performance. Second, financing constraints act as a mediator in the link between executive compensation incentives and firm performance. Third, firm growth moderates the effect of executive compensation incentives on firm performance. Fourth, the positive effect of executive compensation incentives on firm performance is significant in the eastern and central regions, but not in the western region. In the empirical investigation, we apply fixed effects, mediation, and moderation model specifications. To validate the reliability of our findings, we perform additional robustness checks by excluding atypical observations, employing alternative measures of the dependent variable, and adopting an instrumental variable approach. The primary results remain qualitatively unchanged under these alternative settings. Our study enriches the theoretical discourse on executive compensation and firm performance, while also delivering actionable insights for pharmaceutical firms regarding the design of incentive contracts, the alleviation of financing frictions, the adoption of regionally tailored incentive schemes, and the reduction of interregional imbalances. These findings further serve as an empirical foundation and policy reference for relevant stakeholders.

## 1 Introduction

The Chinese pharmaceutical manufacturing industry is currently at a critical juncture of transitioning from scale expansion toward value-driven innovation (1). As healthcare payment reforms, the institutionalization of centralized procurement with volume-based purchasing, and ongoing improvements to the approval pathway for new drugs proceed in tandem, the sector’s competition structure has been radically transformed—excess profitability of generics has been drastically reduced, homogeneous products are exiting the market at an accelerated pace, while novel therapeutics with distinct clinical advantages enjoy both enhanced regulatory encouragement and favorable market valuation. Amid this structural realignment, the survival of pharmaceutical firms is no longer a function of extensive marketing coverage or sheer production capacity, but instead hinges on an integrated set of core competencies—specifically, the scientific planning of R&D pipelines, the efficient steering of clinical development, and the precise mapping of commercialization trajectories (2). In essence, the quality of these strategic decisions rests on the firm leadership’s capacity for information processing, its risk assessment orientation, and its commitment to long-term investment. Against this backdrop, designing an executive incentive system that aligns with the long cycle, high risk, and knowledge-intensive characteristics of the pharmaceutical industry has become a critical governance challenge that directly bears on firms’ core competitiveness.

The operating environment of pharmaceutical firms exhibits several distinctive features that warrant particular attention when compared with that of other industries. Specifically, the pharmaceutical manufacturing sector is characterized by high R&D intensity, elevated risk exposure, and extended development cycles (3). First, the return cycle for innovation is subject to a high degree of uncertainty. From target validation to regulatory approval, the development of an innovative drug encompasses preclinical research, multiple phases of clinical trials, regulatory submission, and other interrelated steps. Failure at any stage along this continuum can render the substantial upfront investments irretrievably lost. This irreversibility of investment and the probabilistic nature of returns compel executives to confront sharper intertemporal trade-offs in resource allocation decisions than their counterparts in other industries. Reducing R&D expenditure can swiftly improve current accounting profits, yet it effectively undermines the long-term viability of the firm. Conversely, sustained increases in R&D investment, while aligning with the firm’s long-term interests, may come at the expense of short-term performance, thereby adversely affecting executives’ personal compensation evaluations and professional reputations. Furthermore, the external financing environment poses rigid constraints on operational decisions. The persistent need for large-scale R&D funding exposes firms to considerable financing constraints; the uncertainty surrounding R&D results and market authorization markedly heightens operational risks; and the lengthy industrialization cycle readily fosters managerial short-termism, which in turn undermines long-term value creation (4–6). The intellectual property and technological know-how accumulated through R&D investment can scarcely serve as effective collateral in credit markets. Together with the pronounced information asymmetry inherent in the R&D process, this imposes more stringent conditions on pharmaceutical firms than on general manufacturing industries in both debt and equity financing channels. When internal operating cash flows fall short of sustaining the R&D pipeline, firms are left with two unpalatable alternatives: either to curtail or even terminate promising projects under development, or to bear prohibitively high financing costs—either course of action ultimately erodes the firm’s long-term performance base. Third, the differentiation among firms in terms of growth stages and development trajectories is particularly pronounced. High growth firms typically enjoy greater room for expansion, stronger resource allocation capabilities, and more stable earnings expectations, which can effectively amplify the positive effect of compensation incentives on firm performance. In contrast, low growth firms, constrained by insufficient growth momentum and limited resources, tend to weaken the transmission efficiency of such incentives (7, 8). The interplay of the above three distinctive features renders the executive incentive issues in the pharmaceutical manufacturing industry both industry specific in nature and embedded with universal theoretical concerns in firm governance research.

Regarding the relationship between executive compensation incentives and firm performance, the academic literature has accumulated considerable theoretical resources and empirical evidence. Employing principal agent theory as the fundamental analytical framework, early studies predominantly approached the issue from the perspectives of compensation level and compensation structure, examining the effects of various incentive instruments—such as monetary compensation, equity incentives, and stock option plans—on firm investment efficiency, innovation output, and financial performance (9, 10). The introduction of tournament theory, in turn, extended the analytical scope from absolute compensation levels to relative pay disparities, revealing the competitive incentive effects inherent in internal pay hierarchies (11). At the empirical level, most studies based on samples of Chinese A-share listed companies report a positive association between compensation incentives and firm performance. However, considerable divergence remains in the findings with respect to industry heterogeneity, variations in incentive instruments, and condition dependent effects of such incentives. Of particular note is that in recent years, some scholars have incorporated financing constraints into the analytical framework of compensation incentives, finding that an increase in incentive intensity helps improve the corporate information environment and strengthen the confidence of external capital providers, thereby alleviating, to a certain extent, the bottlenecks in corporate capital acquisition (12, 13); Other studies have focused on the moderating role of firm growth prospects as a contextual condition, suggesting that firms at different growth stages exhibit systematic differences in their elasticity of response to incentive signals (14). Nevertheless, the mediating role of financing constraints and the moderating role of firm growth have largely been examined in isolation from each other, with few studies integrating both into a unified analytical framework to assess the impact of compensation incentives on firm performance. Moreover, existing studies tend to select samples comprising either all A-share listed companies on the Shanghai and Shenzhen stock exchanges or the broader manufacturing sector, while dedicated, in-depth empirical investigations focusing specifically on the pharmaceutical manufacturing industry—a notably innovation intensive sector—remain relatively scarce. The structural characteristics of this industry, including lengthy R&D cycles, high financing dependence, and uneven regional distribution, have not been adequately captured in prevailing model specifications. With respect to regional heterogeneity, China’s eastern, central, and western regions differ markedly in financial deepening, marketization, and industrial supporting capacities; however, the existing literature has rarely examined, in the context of the pharmaceutical industry, how such regional institutional disparities moderate the ultimate effectiveness of compensation incentives.

Accordingly, the present study focuses on the following three progressively structured core questions: First, does executive compensation incentive have a significantly positive impact on the firm performance of listed pharmaceutical manufacturing firms in China? Does this relationship remain robust after controlling for firm-specific fundamentals and two-way fixed effects? Second, does financing constraints play a mediating role between executive compensation incentives and firm performance? Do executive compensation incentives improve firm performance by alleviating external financing constraints? Third, does firm growth positively moderate the aforementioned relationship? Specifically, can high growth firms more effectively absorb and translate the managerial impetus released by compensation incentives, thereby amplifying the performance enhancing effects of such incentives? Meanwhile, this paper will also examine whether significant regional heterogeneity exists between the eastern/central regions and the western region. Through a systematic examination of the above questions, this study aims to make marginal contributions in the following respects: in terms of industry focus, it compensates for the paucity of dedicated empirical research on executive incentives in the pharmaceutical manufacturing industry; in terms of mechanism identification, it integrates the mediating role of financing constraints and the moderating role of firm growth to construct a more explanatory theoretical framework; and in terms of practical value, it provides empirically grounded decision-making references for the differentiated design of compensation contracts in pharmaceutical firms and for regionally coordinated development policies.

## 2 Theoretical analysis and research hypothesis

### 2.1 Executive compensation and firm performance

Under the modern firm system, the separation of ownership and control constitutes a fundamental feature of firm governance. Shareholders, as owners of the firm, hold residual claims, while management, as the actual executors of operational decisions, exercises day-to-day control over the firm. This separation of ownership and control inherently creates a divergence of interests between principals and agents. Jensen and Meckling, in their seminal work on principal-agent theory, posited that due to information asymmetry and misaligned objective functions between shareholders and management, managers may be motivated to pursue their own self-interest at the expense of shareholder-value maximization, giving rise to agency costs such as excessive perquisites, risk aversion, and underinvestment (15). Consequently, designing an effective incentive mechanism to align the interests of both parties and mitigate agency costs lies at the core of firm governance.

Executive compensation incentives serve as an important institutional arrangement for alleviating the principal-agent problem. According to optimal contracting theory, closely aligning executive compensation with firm performance can induce managers to pursue shareholder value maximization while simultaneously advancing their own interests. Executive compensation incentives represent a core mechanism for reconciling the interests of shareholders and executives and for enhancing firm governance efficiency; they influence executive decision-making through various incentive forms and compensation structures, thereby affecting firm performance. Research on the relationship between executive compensation and firm performance was first conducted by Taussig and Barker, who found only a weak correlation between the two (16). Tournament theory, formally proposed by Lazear and Rosen, places particular emphasis on the incentive value inherent in internal pay dispersion. Reasonable pay differentials across executive tiers can be viewed as a “tournament prize” that effectively stimulates competitive awareness among management. For managers whose compensation levels are relatively disadvantaged, the prospect of attaining higher positions and greater remuneration motivates them to increase work input and intensify effort, thereby fostering a healthy competitive atmosphere within the team. Such managers also become more willing to undertake operational decisions involving a certain degree of risk, ultimately exerting a positive influence on firm performance (11). Qiu and Li (17) using panel data from 332 listed firms over the period 2008–2017, empirically confirmed that executive compensation incentives may promote firm performance. Wang and Wang examined whether executive compensation affects financial performance in state-owned firms, finding that increasing compensation for executives in such firms significantly improves financial performance, and that an appropriate expansion of the pay gap between executives and ordinary employees in state-owned enterprises enhances financial performance and overall firm development (18). Lin et al., using data from Chinese GEM-listed firms, investigated the effect of executive compensation on firm performance and reported a significant positive correlation between the two (19). Zhang et al. argued that compensation incentives can encourage executives to actively engage in risk sharing, thereby strengthening their capacity for risk identification and response, ultimately yielding mutually beneficial outcomes for executive income growth and firm value appreciation (20). Domestic scholars Liu and Sun, using a sample of state-owned listed firms, corroborated a significant positive relationship between internal pay dispersion and firm performance (21); Lin et al. similarly found that pay gaps within top management teams enhance firm performance, providing empirical support for the applicability of tournament theory in China’s capital market (22).

As a quintessentially knowledge-intensive and innovation-driven industry, the pharmaceutical manufacturing sector operates under conditions of substantial R&D outlays, extended payback periods, and pronounced uncertainty. In this context, the quality of managerial strategic decisions—particularly those concerning R&D pipeline configuration, the pace of clinical trial advancement, and the selection of commercialization pathways—directly shapes the firm’s resource allocation efficiency and its capacity for long-term value creation. Effective compensation incentives can motivate executives to proactively enhance their capabilities, orient their efforts toward the firm’s sustainable development, and fully leverage the increasing marginal returns to human capital, thereby providing a robust foundation for sustained performance improvement. Accordingly, this paper proposes the following hypotheses:

H1: *Executive compensation incentives are significantly and positively associated with the performance of pharmaceutical firms.*

### 2.2 The mediating role of financing constraints

Financing constraints refer to a state in which firms are unable to obtain external funds at a reasonable cost and in a timely manner from capital markets to meet their investment and operational needs, due to factors such as information asymmetry and agency problems. Fazzari et al. proposed the use of investment–cash flow sensitivity as a measure of financing constraints. Owing to information asymmetry, a pronounced gap exists between the costs of internal and external financing. The greater the difficulty and severity of external financing constraints faced by a firm, the higher its dependence on internal cash flows for investment expenditure, and consequently, the stronger the financing constraints and the greater the investment–cash flow sensitivity (23).

The root cause of financing constraints lies in information asymmetry between firms and external capital providers. External investors, unable to fully grasp the firm’s true operational status and the potential value of its investment projects, tend to demand a higher risk premium when supplying funds, or may even outright deny funding. Executive compensation incentives, serving as a critical internal governance device, can mitigate this issue to some degree. First, effective compensation incentives can encourage management to improve information disclosure quality and optimize firm governance structures, thereby reducing the degree of information asymmetry between the firm and external investors. Second, a well-designed compensation contract itself serves as a signal to the external market—indicating that the firm possesses sound governance mechanisms and favorable growth prospects—which helps enhance the confidence of external capital providers such as banks and institutional investors in the enterprise. Still, this signaling effect may overlap with signals generated by firm reputation, analyst coverage and other corporate governance mechanisms, as further discussed in Section 6. Zhang and Guo provide empirical evidence that executive monetary compensation incentives significantly reduce the degree of financing constraints, enabling firms to obtain more bank loans and trade credit. Once financing constraints are alleviated, firms can access external funds at lower costs, thereby overcoming the impediments that capital shortages impose on R&D investment and routine operations, and creating foundational conditions for performance improvement (24). Li, using a sample of A-share listed Specialized and Sophisticated enterprises from 2011 to 2023 and employing fixed effects regression models, empirically examines the alleviating effect of management compensation incentives on financing constraints as well as the moderating role of digital transformation. The study finds that compensation incentives for management in such firms significantly curb the level of financing constraints. By aligning the interests of management with those of owners, these incentives mitigate principal agent conflicts, prompt management to accurately disclose firm performance information, reduce information asymmetry, and simultaneously promote R&D investment and performance enhancement, thereby strengthening both internal financing capacity and external credit availability (25). Hao and Zhang, based on a sample of all A-share listed firms from 2012 to 2022 and employing panel regression models and mediation effect models, empirically examined the impact of executive incentives on firm innovation quality and the mediating role of financing constraints. Their findings indicate that both executive compensation incentives and equity incentives significantly enhance innovation quality, and that compensation incentives effectively alleviate financing constraints, thereby providing financial support for innovation activities (26). Liu and Hu, using A-share listed firm data from 2012 to 2022, empirically investigated the effect of executive equity incentives on firm resilience. Their research demonstrates that such incentives effectively alleviate the financing pressures faced by firms, and that the alleviation of financing constraints in turn significantly strengthens firm resilience. This study also provides evidence for the mediating role of financing constraints (27).

For the pharmaceutical manufacturing industry, the alleviation of financing constraints is particularly crucial, as the financing constraints it faces are often more severe than those confronting general manufacturing firms. Executive compensation incentives, by improving the firm’s information environment and governance quality, reduce the information disadvantage and risk concerns of external capital providers, thereby unblocking financing channels. This enables firms to sustain adequate R&D investment and operational flexibility, ultimately driving performance improvement. Accordingly, this paper proposes the following hypothesis:

H2: *Financing constraints play a mediating role in the relationship between executive compensation incentives and pharmaceutical firm performance; that is, compensation incentives indirectly enhance firm performance through the alleviation of financing constraints.*

### 2.3 The moderating role of firm growth

Firm growth denotes the ability of a firm to attain continuous value enhancement in the course of its long-term operations, by steadily optimizing the allocation of both internal and external resources, so as to achieve expansion in scale and improvements in efficiency. As a fundamental indicator of listed firms’ operational performance and developmental prospects, growth is inherently more comprehensive than simple performance measures—it is not confined to a single dimension but embodies a dynamic evolutionary process (28).

Penrose, in her seminal work The Theory of the Growth of the Firm, posited that firm growth is a dynamic process of capability accumulation driven by endogenous resources and constrained by managerial capacity (29), with the dynamic alignment of internal resources and external opportunities jointly facilitating growth. Smith and Watts provide empirical evidence of a significant positive association between managerial equity-based compensation and firm growth (30), suggesting that firms with higher growth prospects are more inclined to adopt incentive-based compensation contracts. In the Chinese context, Xie et al. reported that high-growth firms are better positioned than their low-growth counterparts to implement tournament-based compensation designs (31). Li, drawing on a five-year panel of A-share listed companies (2009–2013), found a significant positive association between firm growth and executive pay. Growth, the study contends, is a direct reflection of managerial effectiveness and a critical yardstick for assessing executive effort and performance. Given that growth signals robust earnings potential, shareholders are thus more inclined to compensate executives in growing listed firms at higher levels, using such pay arrangements as a powerful motivational tool to encourage diligent stewardship (32). Xu and Hou, by examining the moderating role of firm growth on the relationship between innovation input and firm performance, find that the better the firm growth, the stronger the enhancing effect of innovation input on firm performance. Firms with higher growth, owing to their advantages in asset scale, profitability, and market share, are better able to integrate resources and facilitate the implementation of innovation, thereby amplifying the positive impact of innovation input on performance (33).

The reason why high growth firms can amplify the performance enhancing effect of compensation incentives lies in three aspects. First, high growth firms typically enjoy more ample market space and development opportunities, enabling management’s strategic decisions to translate more rapidly into operational outcomes. Second, high growth firms tend to possess stronger resource integration capabilities and capital absorption capacities, which can provide sufficient resource support for the implementation of strategic deployment, thus avoiding the attenuation of incentive effects due to resource bottlenecks. Third, high growth firms have more stable earnings expectations and more optimistic development prospects, which render the incentive signals of compensation contracts clearer and elicit more positive responses from management. Conversely, low growth firms, constrained by structural limitations such as shrinking market space and limited resource endowments, are unlikely to reverse their operational downturns in the short-term even with intensive compensation incentives, rendering the marginal contribution of incentive effects relatively modest. Accordingly, this paper proposes the following hypothesis:

H3: *Firm growth positively moderates the relationship between executive compensation incentives and pharmaceutical firm performance, such that the positive effect of compensation incentives on performance is stronger for firms with higher growth.*

## 3 Research design

### 3.1 Sample selection and data sources

This paper focuses on A-share listed firms in the pharmaceutical manufacturing industry on the Shanghai and Shenzhen stock exchanges over the period 2013–2024. To ensure data reliability and authority, and to prevent outliers from biasing the empirical results, we apply the following screening criteria to the original sample:

(1) We first exclude non-A-share listed entities: firms with stock codes between 200,000 and 300,000 (Shenzhen B-share firms), firms with stock codes between 400,000 and 500,000 (New Third Board/over-the-counter listed firms), and firms with stock codes above 700,000 (including Shanghai B-share firms, rights-issue codes and non-main-board entities). This step retains only A-share listed firms on the Shanghai and Shenzhen main boards, ChiNext and STAR Market;
(2) We retain only observations marked as “normally listed,” thereby excluding firms under special treatment (ST, *ST), particular transfer (PT) or delisting procedures, to avoid interference from abnormal financial and trading statuses;
(3) We exclude firms in the financial industry (classified under category J in the China Securities Regulatory Commission (CSRC) industry classification), and further restrict the sample to firms in the pharmaceutical manufacturing sector (CSRC 4-digit industry code C27), which is fully consistent with the research focus of this paper;
(4) We delete firm-year observations with missing values for any core variable used in the empirical analysis, including executive compensation incentives (Pay), firm performance measures (ROA and ROE), financing constraint index (KZ), firm growth (Growth), and all control variables (Size, Lev, Top10, INST, ListAge, Mshare, and Occupy);
(5) We drop firms with only one firm-year observation over the full sample period, as such singleton observations cannot be effectively identified under the two-way firm and year fixed-effects specification;
(6) To mitigate the influence of extreme outliers and potential data entry errors, all continuous variables are winsorized at the 1st and 99th percentiles, with the winsorized values replacing the original values in all subsequent regression analyses.

After applying the above multi-step screening and cleaning procedures, we obtain an unbalanced panel sample consisting of 2,411 firm-year observations. In addition, all data are obtained from the CSMAR database.

### 3.2 Variables definition

#### 3.2.1 Dependent variable

Firm performance. Firm performance is a composite measure of a firm’s operational achievements and value generating capacity over a fiscal period, reflecting efficiency, profitability, and market standing. Profitability is commonly gauged by ROA, ROE, and operating margin. Following Peng et al., we measure firm performance using ROA (34). For robustness purposes, we also substitute ROA with ROE in alternative specifications.

#### 3.2.2 Independent variable

Executive compensation incentives. Executive compensation incentives is defined as short-term cash compensation awarded to senior management. To address heteroskedasticity, we transform the compensation figures by taking natural logarithms, which also reflects the magnitude and strength of incentive provision. Following Pan, we measure executive compensation incentives as the natural logarithm of the total compensation of the top three executives (35).

#### 3.2.3 Mediating variable

Financing Constraints. Financing constraints capture the predicament in which firms, facing information asymmetries, agency frictions, and transaction costs in capital markets, are precluded from accessing sufficient external funds on a timely basis to sustain their investment and operating activities. Widely adopted measures are the KZ index, the WW index, and the SA index. Following Liu et al., we use the KZ index as our measure of financing constraints. A higher KZ index reflects a greater degree of financing constraints faced by the firm (36).

#### 3.2.4 Moderating variables

Firm Growth. Following Zhou and Wu, we adopt the operating revenue growth rate (37). The growth rate is defined as the change in operating revenue from the prior year to the current year, scaled by the prior year operating revenue. A higher value of this ratio reflects stronger firm growth.

#### 3.2.5 Control variables

This paper includes firm size, leverage, institutional ownership, and other variables as control variables. In addition, we control for year and firm fixed effects. Table 1 summarizes the specific definitions and measurements of the control variables.

**Table 1:** Variable definitions and descriptions.

Variable type	Variable name	Variable symbol	Variable definition
Dependent variable	Firm Performance	ROA	Net income/average total assets
		ROE	Net income/average shareholders’ equity
Independent variable	Executive compensation incentives	Pay	Ln (total compensation of the top three executives)
Mediating variable	Financing constraints	KZ	KZ index
Moderating variable	Firm growth	Growth	(Current revenue—Prior revenue)/prior revenue
Control variables	Firm size	Size	Ln (annual total assets)
	Leverage ratio	Lev	Year-end total liabilities/year-end total assets
	Institutional investor ownership	INST	Institutional shareholding/total shares outstanding
	Ownership share of the top ten shareholders	Top 10	Top 10 shareholders’ shareholding/total shares
	Listing age	ListAge	Ln (current year—listing year + 1)
	Management ownership	Mshare	Directors, supervisors and executives’ shareholding/total shares
	Large shareholder’s fund occupation	Occupy	Net other receivables/total assets

### 3.3 Model construction

#### 3.3.1 Baseline regression model

Following Jensen and Meckling and Murphy, we construct a two-way fixed effects baseline regression model (Model 1) to test the direct effect of executive compensation on pharmaceutical firm performance (15, 38), as shown in Equation 1:


$$\mathrm{RO}A_{i,t}=\alpha_{0}+\alpha_{1}\mathrm{Pa}y_{i,t}+\sum \beta_{n}\text{Controls}+\lambda_{i}+\mathrm{yea}r_{t}+\varepsilon_{i,t}$$ (1)

In the equation, i and t represent the data of firm i in year t. $\alpha_{0}$ *is the intercept;* $\alpha_{1}$ *and* $\beta_{n}$ *are the coefficients of the variables.* $\mathrm{RO}A_{i,t}$ represents firm performance of firm *i* in year *t*; $\mathrm{Pa}y_{i,t}$ represents executive compensation incentives of firm *i* in year *t*; Controls represent the series of control variables described above. $\lambda_{i}$ represents firm fixed effects; $\mathrm{yea}r_{t}$ represents year fixed effects; and $\varepsilon_{i,t}$ is the random error term.

#### 3.3.2 Mediation effect model

Following the mediation effect framework of Wen et al., we specify Model (2), Model (3) *and* Model (4) as presented in Equations 2–4 below to test the mediating role of financing constraints between executive compensation incentives and firm performance (39):


$$\mathrm{RO}A_{i,t}=\alpha_{0}+\alpha_{1}\mathrm{Pa}y_{i,t}+\sum \beta_{n}\text{Controls}+\lambda_{i}+\mathrm{yea}r_{t}+\varepsilon_{i,t}$$ (2)

$$KZ_{i,t}=\alpha_{0}+\alpha_{1}\mathrm{Pa}y_{i,t}+\sum \beta_{n}\text{Controls}+\lambda_{i}+\mathrm{yea}r_{t}+\varepsilon_{i,t}$$ (3)

$$\begin{array}{l} \mathrm{RO}A_{i,t}=\alpha_{0}+\alpha_{1}\mathrm{Pa}y_{i,t}+\alpha_{2}KZ_{i,t}+\sum \beta_{n}\text{Controls}\\ +\lambda_{i}+\mathrm{yea}r_{t}+\varepsilon_{i,t} \end{array}$$ (4)

Model (2) examines the total effect of executive compensation incentives on firm performance. Model (3) investigates the impact of executive compensation incentives on financing constraints. Model (4) incorporates both executive compensation incentives and financing constraints simultaneously to assess the direct and indirect effects.

According to the stepwise approach, a mediation effect is established if the coefficient of executive compensation in Model (2) is significant, and both the coefficient of executive compensation in Model (3) and the coefficient of financing constraints in Model (4) are statistically significant. If the coefficient of executive compensation in Model (4) is not significant, it indicates full mediation; otherwise, it suggests partial mediation. All models include firm-fixed effects and year-fixed effects to control for unobserved heterogeneity and the meanings of the remaining symbols are the same as in Equation 1.

#### 3.3.3 Moderating effect model

To examine whether firm growth moderates the effect of executive compensation incentives on firm performance, a moderation effect model is constructed following Wen et al. (40). Prior to the formation of the interaction term, both executive compensation and firm growth are mean centered to reduce correlation among the variables. Accordingly, the following moderation effect model, Model (5), is specified as shown in Equation 5:


$$\begin{array}{l} \mathrm{RO}A_{i,t}=\alpha_{0}+\alpha_{1}\mathrm{Pa}y_{i,t}+\alpha_{2}\text{Growt}h_{i,t}+\alpha_{3}G\text{rowth}*\mathrm{Pa}y_{i,t}\\ +\sum \beta_{n}\text{Controls}+\lambda_{i}+\mathrm{yea}r_{t}+\varepsilon_{i,t} \end{array}$$ (5)

In the equation, $G\text{rowth}*\mathrm{Pa}y_{i,t}$ is the interaction term between the explanatory variable and the moderating variable. If $\;\alpha_{3}\;$ is significant, it indicates that firm growth significantly moderates the effect of executive compensation incentives on firm performance. In addition, all models include firm-fixed effects and year-fixed effects to mitigate omitted variable bias, and the meanings of the remaining symbols are the same as in Equation 1.

## 4 Empirical results and analysis

### 4.1 Descriptive statistical analysis

As can be seen from the results in Table 2, the mean value of ROA is 0.049, with a standard deviation of 0.077, a minimum of −0.268, and a maximum of 0.243. The mean is smaller than the standard deviation, reflecting that the firm performance of the sample firms exhibits a very high degree of dispersion. The data distribution is highly scattered, with significant differences among individual firms. In addition, the presence of negative values for firm performance indicates that some firms are experiencing losses, while others demonstrate excellent profitability.

**Table 2:** Descriptive statistical analysis.

VarName	Obs	Mean	SD	Min	Max
ROA	2,411	0.049	0.077	−0.268	0.243
Pay	2,411	14.769	0.744	13.137	16.762
KZ	2,411	0.066	2.699	−7.424	6.979
Growth	2,411	0.122	0.350	−0.634	2.114
Size	2,411	22.088	0.963	19.991	24.638
Lev	2,411	0.310	0.173	0.044	0.801
Top10	2,411	0.566	0.144	0.238	0.879
INST	2,411	0.414	0.225	0.012	0.877
ListAge	2,411	2.255	0.767	0.693	3.401
Mshare	2,411	0.140	0.184	0.000	0.642
Occupy	2,411	0.012	0.022	0.000	0.132

The mean value of Pay is 14.769, with a standard deviation of 0.744, a minimum of 13.137, and a maximum of 16.762. The mean is much larger than the standard deviation, indicating that the overall distribution of executive compensation across the sample firms is relatively concentrated, with a low degree of dispersion and relatively moderate differences among firms. The range (max–min) of executive compensation is 3.625. Although there is some interval variation, the overall fluctuation range is limited, with no extremely high or abnormally low values.

### 4.2 Multicollinearity test

Multicollinearity is a common nuisance in multiple regression analysis, as it may inflate the variances of coefficient estimates, invalidate significance tests, and produce erratic coefficient signs. These issues can lead to spurious regression outcomes, thereby compromising the reliability and explanatory power of the empirical results. To ensure the validity of our analysis, we conduct a multicollinearity diagnostic prior to regression estimation. The results are presented in Table 3.

**Table 3:** Multicollinearity test.

Variable	VIF	1/VIF
INST	4.570	0.219
Mshare	3.990	0.251
Top10	3.020	0.331
ListAge	2.130	0.469
Size	2.050	0.487
Lev	1.860	0.538
KZ	1.840	0.545
Pay	1.520	0.656
Occupy	1.130	0.887
Growth	1.070	0.935
Mean VIF	2.320	

The mean VIF for all variables, as presented in Table 3, is 2.320, well below the commonly accepted threshold of 10. Furthermore, all individual VIFs are below 10, indicating that severe multicollinearity is not a concern in our model. This suggests that our model specification is appropriate and suitable for the subsequent regression analysis.

### 4.3 Baseline regression

Based on the baseline regression model constructed in this paper, we conduct the regression analysis and report the results in Table 4. As shown in the table, with the stepwise inclusion of control variables, the r^2^ of the model increases from 0.570 to 0.617, and the adjusted r^2^ rises from 0.502 to 0.554. This indicates that the explanatory power of the model for firm performance is gradually enhanced after adding controls, confirming the soundness of our control variable selection and the controls’ effectiveness in mitigating omitted variable bias.

**Table 4:** Baseline regression.

Variable	(1)	(2)	(3)
	ROA	ROA	ROA
Pay	0.0300***	0.0258***	0.0245***
	(0.00394)	(0.00372)	(0.00368)
Size		0.0232***	0.0264***
		(0.00506)	(0.00521)
Lev		−0.159***	−0.138***
		(0.0160)	(0.0167)
Top10			−0.0932***
			(0.0302)
INST			0.116***
			(0.0222)
ListAge			−0.0376***
			(0.00661)
Mshare			0.0594***
			(0.0217)
Occupy			0.00996
			(0.0816)
_cons	−0.393***	−0.794***	−0.773***
	(0.0581)	(0.120)	(0.120)
Firm	Yes	Yes	Yes
Year	Yes	Yes	Yes
N	2,411	2,411	2,411
F	58***	65***	36***
r2	0.570	0.605	0.617
r2_a	0.502	0.542	0.554

Standard errors in parentheses. *p < 0.1, **p < 0.05, ***p < 0.01.

Table 4 presents the regression results in three columns. Column (1) reports the estimates without any control variables. Column (2) includes a subset of controls, while Column (3) incorporates the full set of control variables. Across all three specifications, executive compensation incentives exhibit a significantly positive effect on firm performance. In the fully specified model of Column (3), the coefficient of executive compensation incentives is 0.0245, significant at the 1% level. This implies that a one unit increase in executive compensation incentives is associated with a 0.0245 unit improvement in firm performance, further corroborating the positive role of compensation incentives in enhancing performance. Thus, our hypothesis H1 is supported.

### 4.4 Robustness test

To test the robustness of our core findings, we construct two robustness check models. These involve, respectively, excluding a specific subsample and replacing the dependent variable with an alternative measure. The regression results are presented in the following table. Specifically, Column (1) reports the regression results after excluding the 2020 COVID-19 subsample, with ROA as the dependent variable. Column (2) presents the results when the dependent variable is replaced by ROE. In addition, both firm fixed effects and year fixed effects are controlled for in the two models. The detailed results are shown in Table 5.

**Table 5:** Robustness test.

Variable	(1)	(2)
	2020 ROA excluded	Dependent variable replaced with ROE
Pay	0.0250***	0.0307***
	(0.00378)	(0.00684)
Size	0.0253***	0.0524***
	(0.00528)	(0.0101)
Lev	−0.140***	−0.265***
	(0.0175)	(0.0397)
Top10	−0.0915***	−0.0954*
	(0.0316)	(0.0567)
INST	0.119***	0.207***
	(0.0234)	(0.0387)
ListAge	−0.0360***	−0.0395***
	(0.00666)	(0.0116)
Mshare	0.0558**	0.108***
	(0.0224)	(0.0389)
Occupy	0.0340	0.0735
	(0.0844)	(0.179)
_cons	−0.761***	−1.425***
	(0.122)	(0.226)
Firm	Yes	Yes
Year	Yes	Yes
N	2,204	2,411
F	34***	22***
r2	1	1
r2_a	0.564	0.483

Standard errors in parentheses. *p < 0.1, **p < 0.05, ***p < 0.01.

As shown in the table, the coefficient of Pay in Column (1), which excludes the 2020 pandemic subsample, is 0.0250. In Column (2), where ROE is used as an alternative dependent variable, the coefficient of executive compensation incentives is 0.0307. Across both robustness models, the core explanatory variable yields positive coefficients that are significant at the 1% level. This indicates that the positive performance effect of compensation incentives is robust and is not driven by sample selection or the choice of performance measure. The two robustness tests together corroborate the stability of our main findings and reinforce the conclusion that executive compensation incentives exert a significant positive effect on firm performance.

### 4.5 Endogeneity test

To account for potential endogeneity in our main independent variable, including reverse causation and omitted variable bias, this paper employs the two-stage least squares (2SLS) method for endogeneity testing. Specifically, we adopt an instrumental variable (IV) approach, using the one period lag of the explanatory variable as the instrument. The regression results are presented in Table 6.

**Table 6:** Endogeneity test.

Variable	(1)	(2)
	2SLS First Stage: Pay	2SLS Second Stage: ROA
Pay		0.0198**
		(0.00797)
IV	0.509***	
	(0.0353)	
Size	0.128***	0.0310***
	(0.0303)	(0.00693)
Lev	−0.0576	−0.158***
	(0.0789)	(0.0189)
Top10	−0.143	−0.0924***
	(0.149)	(0.0343)
INST	0.214*	0.102***
	(0.125)	(0.0251)
ListAge	−0.0222	−0.0466***
	(0.0562)	(0.0100)
Mshare	0.182*	0.0389
	(0.108)	(0.0242)
Occupy	−0.532	0.0463
	(0.330)	(0.0904)
Firm	Yes	Yes
Year	Yes	Yes
N	2026	2026
F	38***	21***
r2	0.879	0.135
r2_a	0.858	−0.018

Standard errors in parentheses. *p < 0.1, **p < 0.05, ***p < 0.01.

The results reported in the table indicate that, in the first stage regression of the 2SLS estimation, the coefficient on the instrumental variable (IV) is 0.509, significant at the 1% level. This suggests a strong correlation between the IV and the core explanatory variable, satisfying the relevance condition of the instrument, and confirming its effectiveness in explaining variations in executive compensation incentives. The corresponding F statistic of 38, far exceeding the conventional threshold of 10, further rules out the concern of weak instruments. In the second stage regression, the coefficient on the core explanatory variable is 0.0198 and remains statistically significant at the 5% level. This indicates that, even after accounting for potential endogeneity, executive compensation incentives continue to exert a significant positive effect on firm performance, consistent with the baseline findings. These results further validate the robustness of our main conclusion and suggest that endogeneity bias is not a serious concern in our analysis.

## 5 Further analysis

### 5.1 Mediation effect test

Based on the mediation effect model constructed in this paper, we conduct the mediation analysis and report the results in Table 7. As shown in the table, Column (1) reports the total effect of executive compensation incentives on firm performance. The coefficient of executive compensation incentives is 0.0245, which is statistically significant at the 1% level. This indicates that a one unit increase in compensation incentives leads to a significant improvement in firm performance of approximately 0.0245 units, confirming that the total effect is significant. Column (2) reveals that the coefficient on executive compensation incentives is −0.560, statistically significant at the 1% level, implying that executive compensation is significantly negatively associated with financing constraints. In other words, enhanced compensation incentives tend to reduce financing constraints. When financing constraints are included as a mediator, the coefficient of executive compensation incentives is 0.0167 and remains significant at the 1% level, confirming that the positive effect of compensation on firm performance persists. Furthermore, financing constraints are found to exert a significantly negative effect on firm performance. Based on the above regression results, it can be concluded that the mediation effect is established, and thus hypothesis H2 is supported.

**Table 7:** Mediation effect test.

Variable	(1)	(2)	(3)
	ROA	KZ	ROA
Pay	0.0245***	−0.560***	0.0167***
	(0.00368)	(0.123)	(0.00319)
KZ			−0.0139***
			(0.000848)
Size	0.0264***	−1.415***	0.00677
	(0.00521)	(0.153)	(0.00486)
Lev	−0.138***	8.185***	−0.0244
	(0.0167)	(0.368)	(0.0173)
Top10	−0.0932***	1.052	−0.0786***
	(0.0302)	(0.822)	(0.0287)
INST	0.116***	−0.423	0.111***
	(0.0222)	(0.622)	(0.0213)
ListAge	−0.0376***	2.043***	−0.00925
	(0.00661)	(0.230)	(0.00591)
Mshare	0.0594***	0.698	0.0691***
	(0.0217)	(0.599)	(0.0199)
Occupy	0.00996	2.759	0.0483
	(0.0816)	(1.986)	(0.0770)
_cons	−0.773***	31.90***	−0.330***
	(0.120)	(3.524)	(0.109)
Firm	Yes	Yes	Yes
Year	Yes	Yes	Yes
N	2,411	2,411	2,411
F	36***	105***	63***
r2	0.617	0.740	0.678
r2_a	0.554	0.698	0.625

Standard errors in parentheses. *p < 0.1, **p < 0.05, ***p < 0.01.

### 5.2 Moderation effect test

Based on the moderation effect model constructed in this paper, we conduct the moderation analysis by creating an interaction term between firm growth and the explanatory variable. The regression results of the moderation effect are reported in Table 8. As presented in the table, the estimated coefficient of the interaction term Growth_Pay_c is 0.0126, statistically significant at the 1% level. The sign of this coefficient aligns with that of the baseline estimate for executive compensation incentives, and both are significant, indicating that firm growth positively moderates the relationship between compensation and performance. That is, the performance enhancing effect of compensation incentives becomes more pronounced as firm growth improves, while it diminishes when growth is lower. Thus, hypothesis H3 is supported.

**Table 8:** Moderation effect test.

Variable	(1)
	ROA
Pay	0.0203***
	(0.00329)
Growth_Pay_c	0.0126***
	(0.00462)
Growth	0.0566***
	(0.00458)
Size	0.0228***
	(0.00494)
Lev	−0.149***
	(0.0157)
Top10	−0.0894***
	(0.0286)
INST	0.0932***
	(0.0211)
ListAge	−0.0289***
	(0.00602)
Mshare	0.0482**
	(0.0201)
Occupy	0.0444
	(0.0770)
_cons	−0.643***
	(0.111)
Firm	Yes
Year	Yes
N	2,411
F	44***
r2	0.665
r2_a	0.610

Standard errors in parentheses. *p < 0.1, **p < 0.05, ***p < 0.01.

### 5.3 Heterogeneity analysis

To investigate potential regional heterogeneity in the effect of executive compensation on firm performance, we partition the sample into eastern central and western regional subsamples and estimate separate regressions, with the results shown in Table 9. The table shows that the effect of executive compensation on firm performance varies significantly across regions: it is significantly positive in the eastern and central regions, but becomes insignificant in the western region. The table reveals that, for the eastern and central regions, the estimated coefficient of executive compensation incentives is 0.0277, statistically significant at the 1% level, suggesting a strong positive effect on firm performance. This result is likely driven by the mature market conditions, sufficient resource endowments, and effective governance structures in the eastern central areas, which enable compensation incentives to be more efficiently converted into performance gains. By contrast, the western region exhibits a lower level of marketization and greater market barriers compared with the well-established market systems in the eastern and central regions. The empirical findings indicate that, in the western region, governance variables such as institutional ownership and management shareholding are insignificant, implying inefficient resource allocation and weak firm governance. This likely impedes the transmission of compensation induced incentives to firm performance through the market mechanism. Moreover, the western region is marked by a less advanced industrial structure and inferior development quality. The industrial makeup is relatively undiversified, largely consisting of resource based and primary processing sectors that feature low value added, limited competitiveness, inadequate R&D expenditure, and serious product homogeneity. This structural pattern tends to depress operational efficiency, thereby hindering the transmission of compensation incentives into firm performance via industrial upgrading or technological progress.

**Table 9:** Heterogeneity analysis.

Variable	(1)	(2)
	East-Central ROA	Western ROA
Pay	0.0277***	0.00626
	(0.00408)	(0.00930)
Size	0.0225***	0.0306**
	(0.00571)	(0.0153)
Lev	−0.147***	−0.194***
	(0.0190)	(0.0393)
Top10	−0.0687*	−0.0467
	(0.0356)	(0.0729)
INST	0.106***	0.0649
	(0.0263)	(0.0518)
ListAge	−0.0393***	0.00832
	(0.00755)	(0.0152)
Mshare	0.0473*	0.0637
	(0.0245)	(0.0610)
Occupy	0.0473	0.0652
	(0.101)	(0.168)
_cons	−0.737***	−0.680*
	(0.130)	(0.355)
Firm	Yes	Yes
Year	Yes	Yes
N	1977	343
F	32***	5***
r2	0.633	0.554
r2_a	0.571	0.452

Standard errors in parentheses. *p < 0.1, **p < 0.05, ***p < 0.01.

## 6 Conclusions and recommendations

Based on a sample of A-share listed pharmaceutical manufacturing companies in China, this study empirically tests the impact of executive compensation incentives on firm performance, and delves into the mediating effect of financing constraints and the moderating effect of firm growth. A regional heterogeneity analysis is also conducted. The main findings are summarized as follows. First, a significantly positive relationship is identified between executive compensation incentives and the performance of pharmaceutical firms, suggesting that increased executive pay can effectively motivate management, refine decision-making and resource allocation, and promote efficiency and profitability improvements. Second, financing constraints are found to play a significant mediating role in the relationship between compensation incentives and firm performance; compensation incentives significantly alleviate financing constraints, which in turn enhances performance. Third, a significant positive moderating effect of firm growth is confirmed, indicating that high growth firms tend to magnify the positive effect of compensation incentives on performance. Fourth, significant regional heterogeneity is observed: the positive effect of compensation incentives is robust in the eastern and central regions, but is not statistically significant in the western region, where the incentive effect is difficult to convert into improved operating performance. Despite consistent evidence that executive compensation incentives boost pharmaceutical firms’ performance, several competing mechanisms and potential downsides should be noted when interpreting our results. First, though firm size is controlled in all regressions, the positive link cannot be fully ascribed to incentive effects. It could stem from alternative channels: talented managers command higher pay and deliver better performance; larger firms or those in highly-concentrated industries can afford generous pay while possessing intrinsic performance advantages. Our results reflect the average treatment effect conditional on controls, yet data limitations prevent us from cleanly separating incentive effects from selection effects related to managerial talent, firm scale and market power. Second, as discussed in Section 2.2, well-designed compensation contracts send positive signals to external capital markets. However, this signaling effect may be confounded by firm reputation, analyst coverage and other governance mechanisms, which can shape market perceptions independently of compensation. Our study cannot isolate the pure signaling effect of compensation contracts. Third, given the high uncertainty of pharmaceutical R&D, strong compensation incentives may trigger risk-shifting behavior. Executives may pursue excessive, value-destroying risky projects: they gain from successful gambles, while shareholders bear most downside losses. This paper focuses on the average net performance effect and does not test these adverse risk-taking outcomes. Based on the empirical findings, this paper proposes the following recommendations:

First, optimize the executive compensation incentive system and strengthen the long-term incentive orientation while guarding against potential adverse risk-shifting consequences. A diversified compensation system that is closely tied to performance should be established by pharmaceutical firms. Firms should set reasonable rather than excessive incentive intensity and pay close attention to the rationality of internal pay gaps, so as to balance incentive effectiveness, risk control and internal fairness. The tournament incentive effect may thus be activated to enhance managerial motivation and competitiveness, without encouraging value-destroying excessive risk-taking in R&D and investment decisions. Furthermore, the transparency of compensation design, appraisal, and payment should be improved, and firms should be subject to oversight by shareholders, regulators, and the public, thereby ensuring the fairness and effectiveness of the compensation mechanism.

Second, alleviate financing constraints for pharmaceutical firms and unblock the transmission channels of incentives. To reduce financing constraints for pharmaceutical firms, actions should be taken at three levels: the firm, financial institutions, and government policy. At the firm level, the governance benefits of executive compensation should be used to improve governance, disclosure quality, and information transparency. At the financial institution level, banks, securities, and insurance firms should offer innovative financial products targeting the unique needs of pharmaceutical firms to ease financing access. At the government level, financing support policies should be expanded and strengthened, with more resources directed towards pharmaceutical innovation to effectively ease constraints.

Third, implement differentiated incentive strategies based on firm growth. High growth pharmaceutical firms should moderately increase compensation incentive intensity to encourage R&D innovation and market expansion, unlocking the synergy between incentives and high growth. Low growth firms, by contrast, should strengthen their growth base through product mix improvement, cost reduction, and niche market expansion, while adopting more targeted and binding incentive schemes, with intensity gradually optimized as growth picks up. Overall, firms should dynamically align incentive intensity and structure with their developmental stage to enhance relevance and effectiveness.

Finally, narrow the regional development gap and enhance the effectiveness of incentives in the western region. Efforts are needed in two directions. First, marketization mechanisms in the western region should be accelerated, market-based resource allocation improved, internal governance refined, and managerial behavior regulated, to remedy governance deficiencies and provide a supportive institutional environment for compensation incentives. Second, coordinated development of the pharmaceutical industry should be promoted, with innovation resources more effectively channeled to the west to upgrade its industrial structure and innovation capacity, thereby enabling the full realization of incentive effects and gradually narrowing the regional development gap.

## 7 Limitations and future research

This study has two main limitations. First, while we test the mediating role of financing constraints and the moderating role of firm growth, the effect of financing constraints may vary across firm life cycle stages (growth, maturity, and decline). Second, this study aggregates all pharmaceutical manufacturing firms into a single sample. Sub-sectors including biotechnology, chemical drugs, traditional Chinese medicine, and medical devices exhibit substantial differences in R&D intensity, regulatory pathways, and business models. As this paper does not split the sample by these sub-sectors, the estimated coefficients should be interpreted as the overall average effect for pharmaceutical manufacturing firms and do not imply that the incentive-performance relationship is homogeneous across all sub-sectors. Future research may conduct sub-sector heterogeneity analyses to test whether industry-specific R&D features moderate the relationship between executive compensation incentives and firm performance. Third, while firm size is controlled for in our models, our dataset lacks direct proxies for managerial ability, industry concentration, firm reputation and analyst coverage. Therefore, we cannot empirically disentangle our hypothesized incentive mechanism from competing explanations based on managerial talent, market power and alternative market-signaling channels. In addition, we are unable to empirically examine the risk-shifting problem, i.e., excessive value-destroying risk-taking triggered by high executive compensation in high-R&D pharmaceutical firms. Future research may incorporate these variables to distinguish multiple competing mechanisms and assess both the beneficial and adverse impacts of executive compensation incentives.

## Data Availability

The firm‑level panel data that support the findings of this study are obtained from the proprietary CSMAR database. Restrictions apply to the availability of these data due to CSMAR’s commercial‑use license agreement; therefore, the raw underlying dataset cannot be publicly deposited. Qualified researchers who purchase legal access to the CSMAR database can reproduce the empirical results following the sample‑screening rules, variable definitions and model specifications reported within this manuscript. The analysis code used in this study is available from the corresponding author upon reasonable request.
